# Justice at the interface: advancing community and health system resilience through intersectionality theory

**DOI:** 10.1093/heapol/czag005

**Published:** 2026-01-19

**Authors:** Jen Roux, Neelke Doorn, Saba Hinrichs-Krapels, Samantha Copeland

**Affiliations:** Faculty of Technology, Policy and Management, Delft University of Technology, Jaffalaan 5, Delft 2628 BX, The Netherlands; Faculty of Technology, Policy and Management, Delft University of Technology, Jaffalaan 5, Delft 2628 BX, The Netherlands; Faculty of Technology, Policy and Management, Delft University of Technology, Jaffalaan 5, Delft 2628 BX, The Netherlands; Faculty of Technology, Policy and Management, Delft University of Technology, Jaffalaan 5, Delft 2628 BX, The Netherlands

**Keywords:** health system resilience, community, social justice

## Abstract

Current approaches to health system resilience tend to prioritize system-level outcomes (e.g. functionality) while overlooking key underlying social processes, contexts, and power-laden interactions through which resilience is produced. When community resilience is subsumed under health system resilience, without attending to distinct contextual factors, it can lead to fragmented approaches or maladaptive outcomes that misalign with the resilience of communities. Therefore, resilience approaches need to include additional methods that incorporate analyses of power structures and context. We propose intersectionality theory as a methodological lens to investigate the underlying social processes and power dynamics that shape community resilience and health system resilience interactions. An intersectionality approach prompts researchers to distinguish how resilience capacity is derived through the involvement of community actors, their unique intersecting social identities, and their lived experiences. Including an intersectional lens in resilience approaches provides researchers with the tools to identify points of practical constraints that arise at the intersection of communities and health systems, with particular attention on the burdens that are placed on community actors.

Key messages **Methodological Gap**

- Resilience approaches are being increasingly adopted for capacity building in health systems and communities. While interrelated, community resilience is not synonymous with health system resilience. Current approaches tend to focus on system-level outcomes, while failing to capture the complex social processes and interactions through which resilience is produced at the community level.
- In practice, this leads to a reliance on the community without providing adequate support or resources. Further, there exists little methodological guidance on how to capture the complex interaction between community and health system resilience. We propose intersectionality theory as methodological lens to navigate practical and equity-related resilience challenges that emerge between communities and health systems.

**Methodological development**

- Intersectionality draws specific attention to power dynamics and contextual realities. Methodologically, intersectionality theory prompts researchers to distinguish how resilience capacity emerges through the involvement of community actors, their unique intersecting social identities, and their lived experiences. Developing resilience methodologies to incorporate analyses of power and context reveals how and why resilience capacities are developed, for whom, and under what conditions.

## Introduction

Health system resilience (HSR) has become an increasingly prominent focus in global health discussions. However, any meaningful discussion of resilience must begin with a clear sense of who constitutes the health system and how different actors contribute to its capacities. Kruk et al. (2015) defined HSR as *‘*the capacity of health actors, institutions, and populations to prepare for and effectively respond to crises; maintain core functions when a crisis hits; and, informed by lessons learned during the crisis, reorganize if conditions require it’. The resilience of health systems can then be appropriately characterized by their connectivity between and within other complex subsystems, including communities (Karamagi et al. 2022, Witter et al. 2023, Copeland et al. 2023).

It follows, therefore, that many conceptualizations of HSR encompass community resilience (CR), focusing on multiple actors and domains beyond the formal health sector (Alameddine et al. 2019, South et al. 2021, World Health Organization 2022, Than et al. 2024). These include informal health actors such as individuals, communities, families, local organizations, and alternative providers who are not institutionally or formally regulated but still provide and influence care (Aldrich and Meyer 2015, Saulnier et al. 2021 , Grimm et al. 2022, Chumo et al. 2023, Kwamie et al. 2024). Here, this framing is used heuristically, as distinctions between formal and informal actors are context-specific and vary across settings. These actors contribute essential support, local knowledge, and social cohesion and can self-mobilize in response to acute shocks and everyday stressors (Jewett et al. 2021, Chumo et al. 2023, Shirleyana et al. 2023). Thus, CR research within the context of health systems has important implications for the effectiveness and adaptability of healthcare services (Bhandari and Alonge 2020).

While HSR and CR are interrelated, the resilience of the health system is not synonymous with the resilience of the community. Current approaches to HSR tend to prioritize system-level outcomes (e.g. functionality) while overlooking underlying social processes, contexts, and power-laden interactions through which resilience is produced (Chaplin et al. 2019, Ramcilovic-Suominen and Kotilainen 2020). These factors are critical to understanding the diverse community conditions that resilience measures need to translate into and how various actors involved enact resilience capacities and to avoid unintentionally shifting burdens to marginalized or vulnerable actors (Garcia et al. 2022, Blaas et al. 2025). This presents a methodological challenge, as there is limited guidance on how to capture the complex interactions and relationships linking HSR and CR.

We offer intersectionality theory as a methodological lens for considering the practical and equity-related resilience challenges that emerge between communities and health systems. Intersectionality draws specific attention to how overlapping social systems (e.g. sexism or racism) and different socio-demographic identities (e.g. race, gender, class, sexuality, or ability) interact to shape an individual’s experience of the world (Hankivsky et al. 2014, Bauer and Scheim 2019). The theory further explains why some individuals, given the culmination of their social identities, might experience compounded vulnerabilities that impact their health. In this methodological musing, we suggest that intersectionality theory can advance resilience approaches by illuminating the practical challenges that occur at the interface between CR and HSR. Grounded in a relational understanding of power, intersectionality theory contextualizes how community actors generate capacity for HSR, in relation to their intersecting social identities, and interactions within broader systemic conditions. In the following sections, we first examine the practical challenges of resilience that occur between community and health systems, followed by a discussion of how resilience is differentially constructed and experienced, which prompts the need to further develop current resilience methodologies. We then consider how intersectionality, both as a theory and a method, can usefully expose the power dynamics and capacity inequalities embedded within community and HSR approaches.

## The practical challenges of resilience in communities and health systems

A challenge in translating resilience strategies into practice is that they can often rely on community actors without accounting for the conditions under which they contribute. HSR has been critiqued for assuming systems and communities have a standard baseline capacity—not considering the influence existing inequalities—which may unintentionally shift burdens to marginalized or vulnerable actors (Bhandari and Alonge 2020, Witter et al. 2023, Saulnier and Topp 2024, Blanchet 2025). For example, resilience discourses can miss systemic vulnerabilities by celebrating CR while neglecting the structural conditions that necessitate it or allow health systems to rely on it (Poland et al. 2021). Kaika (2017) highlights this from Tracie Washington’s campaign, ‘Stop calling me resilient’, to illustrate how celebrating communities for their ability to endure, respond to, or bounce back from shocks can obscure the deeper systemic drivers that create or sustain their vulnerability. Similarly, Saulnier and Topp (2024) illustrate how a strategy aimed at increasing adaptive capacity by expanding the health workforce to include a broader range of community health workers (CHWs) can increase HSR. However, the authors note that while this strategy may strengthen HSR, it often does so at the expense of CHWs, impacting their well-being (emotional and physical) and drawing on their personal resources (time and money) and capacities (Ballard et al. 2022, Salve et al. 2023, Saulnier and Topp 2024). Such outcomes only indicate that the system is functioning, but they do not show how it achieves this function, and more critically, at whose expense (Witter et al. 2023). Consequently, community actors who operate at the interface between formal and informal structures tend to mediate this tension (Odii et al. 2024).

Methodologies also need to better account for how resilience is enacted in practice. More specifically, who enacts resilience. This requires an understanding of power dynamics, which essentially determine responsibility arrangements for care, and how actors can access resources or participate in decision-making (Chaplin et al. 2019, Blanchet 2025). For example, during the 2014 Ebola outbreak, the deployment of formal (i.e. institutionally regulated) and informal (i.e. non-mandated) CHWs was seen to be effective in bolstering HSR and increasing community access to services (Miller et al. 2018). In that response, both informal and formal CHWs promoted early disease detection, extended access to health services, and communicated critical health information in a culturally sensitive manner (Miller et al. 2018, Ballard et al. 2022). Both CHWs were positioned within the community in such a way that they were able to extend services and provide key epidemiological and contextual information back to the formal health system. In many communities, families would also first seek out trusted community members (such as informal CHWs) before reporting to a formal health facility (Miller et al. 2018). However, most informal CHWs received little or less access to training and were either unpaid or received lower wages than formal CHWs (Miller et al. 2018, Ballard et al. 2022). Similarly, many informal CHWs—who were women from lower socio-economic backgrounds—expressed that this weak link with the formal health system contributed to power imbalances and a lack of respect from formal CHWs (Miller et al. 2018, Ballard et al. 2022).

In other crisis settings, a similar pattern can be seen where community actors mediate health system gaps. For example, during South Africa’s COVID-19 response, the Community Action Network (CAN) emerged as a self-organized, community-led action group that provided essential care often when the state was unable to or limited capacity to intervene (van Ryneveld et al. 2020). Community networks such as this have been identified as a key mechanism to enhance capacity in HSR and CR, as well as contribute to learning and knowledge sharing in crises (Aldrich and Meyer 2015, Patel et al. 2017, Güngör and Elburz 2024, Odii et al. 2024). The local focus of the CANs enabled the network actors to identify specific socio-economic vulnerabilities, such as food insecurity, and extend neighbourhood-based non-medical support (van Ryneveld et al. 2024). While the CANs formed ‘boundary-spanning’ relationships with health system actors, they received little recognition or material support (van Ryneveld et al. 2020). This placed additional care responsibilities onto CAN actors, some of whom are from vulnerable groups, without the necessary structural support (recognition or resources) from the state (van Ryneveld et al. 2024). The CANs mediated the gap between formal and informal care structures by taking on additional responsibilities to provide vital community-specific support. Both examples, the Ebola CHWs and CANs, show how community actors are necessary for resilience capacities, but also the negative consequences that can occur without explicit consideration of power relations. As the literature on CR has been extended to promote adaptive health systems, so has the responsibility for contributing to resilience capacities onto a broader set of actors (Blanchet 2025). This shift creates a need for resilience methodologies that can support the capacities of these actors, while avoiding undue burdens.

## Rethinking the interface between communities and health systems

Resilience approaches are being increasingly adopted for capacity building in health systems and communities (Blanchet 2025). However, challenges remain in how resilience transitions from conceptualization to practice (Doorn 2017, Sanne et al. 2021, Grimm et al. 2022). The examples above demonstrate the complexities that arise in translating resilience approaches into real-world practice, as well as the significance of the relationship between HSR and CR. Naturally, then, the increasingly recognized contributions of community actors, such as informal health actors (i.e. CHWs or grassroots networks), prompt a practical and conceptual discussion on integrating them into health systems formally (Kumah 2022). However, when the resilience of communities is subsumed under HSR, without attending to contextual realities (i.e. distinct community vulnerabilities, or diverse actor participation), it can lead to fragmented approaches that misalign with the realities of communities. As demonstrated by the examples above, in practice, this may result in maladaptive outcomes or reproduce exploitative relationships, where community capacity is relied upon to compensate for systemic deficiencies or where vulnerable actors shoulder additional responsibilities.

Attending to differences in HSR and CR means being responsive to the way context influences the capacities of communities and health systems. These contextual factors (i.e. cultural, social, political, and environmental elements) shape the capacities of systems and community actors to manage changing conditions, which necessitates resilience strategies that can adapt to diverse contexts (Ramcilovic-Suominen and Kotilainen 2020). For example, while the threat of COVID-19 was shared across the globe, the impact of it differed between and across communities. Long-standing inequities were made evident by disproportionate infection and death rates, and unequal access to healthcare, particularly in marginalized communities and vulnerable populations (Witter et al. 2023, Frey et al. 2024). Community vulnerabilities and their underlying causes impact actor capacities to respond to various shocks and stressors (Chaplin et al. 2019). Structural vulnerability patterns also stem from historical and contemporary patterns of power (Garcia et al. 2022). As demonstrated, these power relations contribute to the degree of influence, priority, accessibility, and visibility specific actors have or do not have (Garcia et al. 2022, Blaas et al. 2025). Developing resilience methodologies to incorporate analyses of power and context is needed to reveal how and why resilience capacities are developed, for whom, and under what conditions (Cutter 2016, Garcia et al. 2022, Witter et al. 2023, Topp 2024).

## Advancing resilience methodologies with intersectionality theory

Intersectionality theory offers resilience researchers a methodological entry point to reveal, analyse, and ultimately challenge the structural conditions that create or uphold different forms of marginalization and privilege. As articulated within the core framework of intersectionality, structural advantages and disadvantages are often co-produced, just as resilience for some comes at the cost of or constraints for others (Doorn et al. 2019, Misra et al. 2021, Saulnier and Topp 2024). Intersectional methodological approaches focus on several key tenets to examine power structures, including relationality, complexity, context, and oppression (Misra et al. 2021). This orientation is particularly effective for resilience research as it directly responds to the methodological gap outlined earlier.

An intersectionality approach prompts researchers to distinguish how resilience capacity is derived through the involvement of community actors, their unique intersecting social identities, and their lived experiences. This situates resilience not as a neutral capacity, but as one that is relational. As previously outlined, while there is often a mutually reinforcing relationship between CR and HSR, this dynamic is not inherently reciprocal (i.e. system functioning does not always equal resilience in communities or positive outcomes for all actors involved). This one-size-fits-all approach exemplifies the intersectionality theory category of ‘single axis’ logic, where outcomes (i.e. ‘the system is functioning’) are viewed in isolation from the intersecting social position and structural conditions that shape how resilience capacities are enacted by different actors (Cho et al. 2013).

Intersectionality embraces complexity and resists ‘flattening’ actor identities or generalizing community actors into monolithic categories. Intersectional scholars would refer to this flattening as ‘additive’ thinking, whereby identities are framed as separate variables that can be ‘added’ together to predict privilege or disadvantage (Hankivsky et al. 2014, Misra et al. 2021). Instead, an intersectional analysis would examine identities multiplicatively, treating them as mutually constitutive, with experiences shaped through their interrelated interactions and context-specific conditions (Hankivsky et al. 2014, Misra et al. 2021). Methodologically, this prompts researchers to ask how categories of difference are interconnected and what practical adjustments need to be made to attend to them. For example, in the CHW case, how formal and informal health actors, their social identities, intersect with structural conditions, such as access to resources, decision-making power and recognition for their contributions (Atewologun 2018, Abrams et al. 2020, Boston et al. 2024). This approach reveals disparate capacity dynamics by attending to actor positionality in relation to the health system, thereby avoiding the flattening of identities and drawing attention to how resilience capacities are shaped within a specific context. It also provides researchers with the tools to identify points of practical constraints that arise, particularly those placed on participating actors, and, importantly, to have a clearer understanding of what is needed to design more equitable resilience strategies.

The strength of intersectionality in resilience lies in its versatility, as it can be applied to any given method. A diverse range of disciplines has leveraged it as guiding theory and framework to challenge conventional or traditional research approaches (Kelly et al. 2021). Contemporary uses of intersectionality have expanded the theory beyond identity categories to apply it to processes and structures (Bauer 2014, Homan et al. 2021). Additionally, intersectional methodological approaches encourage data collection, analysis, and presentation in ways that make visible how multiple, interlocking social identities are situated in relation to socio-economic factors (Bowleg 2012). It has been incorporated across qualitative and quantitative methods to inform all stages of the research process, from conceptualization to design, data collection, analysis, and interpretation (Bauer 2014, Atewologun 2018, Abrams et al. 2020, Misra et al. 2021, Chisty et al. 2021).

A study conducted by Chisty et al. (2021) provides an illustrative example of how intersectionality theory can be applied methodologically. The authors use intersectionality to examine how the interplay of social identities shapes community vulnerabilities and resilience to flood-related events in Bangladesh. Their main goal was to demonstrate the importance of employing an intersectional disaster resilience approach (Chisty et al. 2021). They use a qualitative study design, to collect data on participants’ socio-demographic conditions and intersectional characteristics. Their findings revealed how social identities (particularly marginalized identities, such as being both female and disabled) intersect with structural conditions to create heightened vulnerability during disasters (Chisty et al. 2021). The authors connected intersectional characteristics across various vulnerability categories (social, environmental, economic, cultural, and physical) to illustrate where risks are distributed across the community. Not only were they able to identify where risks and disparities occurred in the community, but they also mapped why they were produced and how resilience strategies can specifically account for them. Overall, through an intersectional methodological approach, they gained a deeper contextual understanding necessary to develop effective and community-responsive disaster resilience strategies.

As demonstrated, while communities are critical drivers of HSR, they often fill gaps in healthcare delivery while navigating systemic deficiencies (Bhandari and Alonge 2020, Chumo et al. 2023, Shirleyana et al. 2023, Witter et al. 2023, Than et al. 2024). Intersectionality theory focuses on ‘centring the margins’, by bringing attention to the experiences of marginalized and vulnerable groups that are often overlooked in dominant paradigms (Homan et al. 2021). This means interrogating the influences on community capacity from a variety of mechanisms, including differential access to resources (e.g. decision-making or health services) and increased exposure to health risks (e.g. social stressors, chronic deprivation, discrimination) (Homan et al. 2021). This approach also incorporates the lived reality of communities and actors within the broader socio-political contexts, historical structural processes, and oppression (Atewologun 2018, Homan et al. 2021). Ultimately, this would advance resilience methodologies to more readily be able to prevent burdening vulnerable actors, as well as creating structural mechanisms to support their participation.

## Conclusion

Kimberlé Crenshaw introduced intersectionality as an approach that highlights where ‘power comes and collides, where it interlocks and intersects’ (Columbia Law School 2017). Inequalities arise from the complex and interconnected interactions of social, economic, political, and environmental systems operating across multiple scales, which produce and reinforce uneven access to resources, opportunities, and power. As inequalities are not experienced uniformly, neither is resilience. The relational and contextual nature of resilience requires methodological approaches that accommodate and attend to differences. Not doing so risks reproducing extractive relationships, relying on community labour and adaptive capacity to compensate for chronic systemic failures. Our contribution addresses this gap by offering a methodological approach, intersectionality theory, that illuminates the complexities of resilience that occur in practice, using the relationship between communities and health systems as a site of exploration.

Intersectionality highlights how and why vulnerability manifests heterogeneously across communities and actors. Methodologically, it helps researchers understand who bears the burden of enacting resilience, under what conditions, and with what consequences. By applying this approach, particularly at the interface of CR and HSR, researchers can move beyond one-size-fits-all models to foreground resilience practices that are adaptive to context and aware of the influence of power relations. Intersectionality would enable resilience strategies to better account for the distinctiveness of these communities and how to support varying capacities, as well as to interrogate the structures and histories that shape resilience outcomes.

## Data Availability

No new data were generated or analysed in this research.

## Reflexivity statement

The authorship team is diverse in terms of nationality, seniority, and disciplinary backgrounds. Our team is composed of four women at various career stages with the first author as a junior researcher. The authors represent a range of nationalities—two from HICs and two from LMICs—and have diverse experiences in topics such as global health, public health, ethics, philosophy, justice, health policy, and resilience domains.
